# Building blocks for automated elucidation of metabolites: Machine learning methods for NMR prediction

**DOI:** 10.1186/1471-2105-9-400

**Published:** 2008-09-25

**Authors:** Stefan Kuhn, Björn Egert, Steffen Neumann, Christoph Steinbeck

**Affiliations:** 1 Leibniz Institute of Plant Biochemistry, Department of Stress and Developmental Biology, Weinberg 3, 06120 Halle, Germany; 2 Cologne University Bioinformatics Center (CUBIC), Zülpicher Str. 47, 50674 Köln, Germany; 3 European Bioinformatics Institute (EBI), Wellcome Trust Genome Campus, Hinxton, Cambridge CB10 1SD, UK

## Abstract

**Background:**

Current efforts in Metabolomics, such as the Human Metabolome Project, collect structures of biological metabolites as well as data for their characterisation, such as spectra for identification of substances and measurements of their concentration. Still, only a fraction of existing metabolites and their spectral fingerprints are known. Computer-Assisted Structure Elucidation (CASE) of biological metabolites will be an important tool to leverage this lack of knowledge. Indispensable for CASE are modules to predict spectra for hypothetical structures. This paper evaluates different statistical and machine learning methods to perform predictions of proton NMR spectra based on data from our open database NMRShiftDB.

**Results:**

A mean absolute error of 0.18 ppm was achieved for the prediction of proton NMR shifts ranging from 0 to 11 ppm. Random forest, J48 decision tree and support vector machines achieved similar overall errors. HOSE codes being a notably simple method achieved a comparatively good result of 0.17 ppm mean absolute error.

**Conclusion:**

NMR prediction methods applied in the course of this work delivered precise predictions which can serve as a building block for Computer-Assisted Structure Elucidation for biological metabolites.

## Background

For successful pharmaceutical treatments the full knowledge about the metabolic and regulatory networks of a system is needed. Only if those pathways are known completely, can we hope to understand how an organism circumvents the blocking of a receptor in one pathway by employing an redundant analog or why a drug not only interacts with its intended target T but also with targets S, I, D and E, thereby creating side effects.

Despite an ever growing number of known gene sequences and elucidated protein structures the knowledge of the molecular components of biological systems remains incomplete. Even for simple model organisms, only 20 – 50% of the metabolite structures are known [1]. For that reason, projects such as the Human Metabolome Project [2] have been started to catalogue the metabolomes of whole organisms.

Nuclear Magnetic Resonance (NMR) is still the only analytical technology capable of giving enough information for elucidating the molecular structure of an unknown substance. This becomes especially important in metabolomics research, where experiments – often measured with the more sensitive mass spectrometry (MS) instruments – detect differential mass signals, but in many cases even tandem MS will not give enough information for a *de-novo *characterisation of the metabolite.

Powerful chromatographic and spectroscopic methods are available to alleviate this lack of knowledge on metabolite structures, but nothing close to an automatic method with high throughput capabilities has yet been developed [3,4]. Systems aimed at doing this are known as Computer Assisted Structure Elucidation (CASE) systems. One way to perform CASE is to generate putative molecules in agreement with certain input data, predict spectra for each of the candidates and rank them by similarity of predicted and experimental spectra. A rudimentary prediction can be done by database lookup, but since these can never contain all possible structures, a prediction for arbitrary structures is needed.

For spectrum prediction of ^13^C and other nuclei, an established method dating back to 1978 are the Hierarchically Ordered Spherical Environment (HOSE) codes [5]. For proton ^1^H NMR spectra more elaborate algorithms have been suggested. This is mainly justified by the strong influence of 3D spatial effects on proton shifts, which are not included in the original HOSE code specification. Therefore methods using only 2D parameters are considered insufficient [6]. Stereochemical enhancements of the HOSE code specification have been described in [7].

The first attempts towards proton NMR predictions were based on string-based descriptions similar to HOSE codes [8]. Commercial packages also use such methods, combined with other strategies [[9,10], p. 80]. Later so-called additivity rules were established, which are manually created rules describing the effect of certain functional groups. They were motivated mainly by the lack of sufficiently large databases, which are needed for string-based descriptions [11]. Finally machine learning methods, most prominently neural networks, were used [9,12-14].

The problem of predicting spectra for arbitrary compounds based on a collection of samples is a classic application of machine learning methods. By including descriptors reflecting distances in Euclidean space, stereochemical information can be included easily by these methods. In this paper, we perform a comparative study of selected methods for the purpose of proton NMR spectrum prediction, we also include HOSE-code based prediction for comparison.

## Methods

In this section we first give a formal definition of the prediction problem, then we describe the test- and training set and the descriptors used to evaluate the machine learning algorithms.

**Figure 1:**
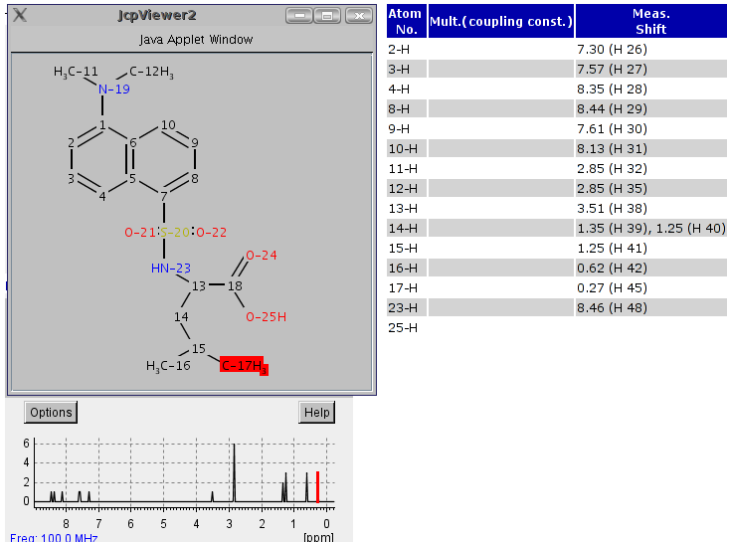
An example of an assigned proton spectrum from the NMRShiftDB (molid 10019871).

An NMR spectrum can be modelled as a collection of individual shifts of atoms. Figure 1 shows a graphical representation of a molecule, the corresponding ^1^H spectrum with the individual peaks and the assignment between shifts and atoms.

In machine learning algorithms, the input is given as feature vector representing the properties of the atom ("descriptors"), and the output is either the target class (classification) or a numerical target value (regression).

The mean absolute error (MAE) and the standard error (SE) of the prediction are calculated as:

$$MAE=\frac{\sum \left|\delta_{exp}-\delta_{pred}\right|}{n}$$

$$SE=\sqrt{\frac{\sum {\left(\delta_{exp}-\delta_{pred}\right)}^{2}}{n}}$$

with *δ*_*exp *_being the experimentally observed chemical shift of an atom, *δ*_*pred *_the predicted chemical shifts of the same atom and *n *the number of data points. To estimate the generalisation error, we performed a 10 fold cross validation by first applying a random permutation to the data set, then dividing the data set into 10 equally sized disjunct partitions in terms of the total numbers of molecules and in each iteration predicted one of the disjunct partitions by the complementary remaining 9 partitions in each iteration, constituting always an overlapping yet different training set. Attention has to be paid to ensure that all protons of the molecule under prediciton are excluded from the training set. Otherwise, shifts would be predicted based on inference from neighbouring protons, resulting in overfitting.

In order to compute the MAE and SE errors for the classification tasks, the response variable (shift in ppm) was discretised into bins of size 0.1 ppm, resulting in 107 different classes ([0..10.7] ppm) (see Table 1).

**Table 1:** Mean absolute error in ppm from a decision tree prediction by proton category classes.

	aromatic *π *systems	non aromatic-*π *systems	rigid aliphatic systems	non rigid aliphatic systems
number of protons	4013	1279	5062	8318
mean abs. error	0.168	0.273	0.204	0.135

### Molecular and atomic descriptors

The proper choice of descriptors has a major influence on the performance. To capture dependencies between the shifts, the protons have to be described together with their chemical context in the molecule. Descriptors can be derived from atomic, bonding, and molecular properties and should capture the atom and its environment at different levels of structure resolution in addition to diverse physicochemical properties of a compound.

416 descriptors in total were calculated using the QSAR (Quantitative Structure Activity Relationship) package of the CDK (Chemistry Development Kit) [15,16]. This package implements a wide range of molecular and atomic QSAR descriptors.

For our study we decided to combine the descriptors from a study by Meiler, who used Artifial Neural Networks to predict chemical shifts in proteins. [12] with most of those from work by Aires-de-Sousa [9]. From [12] we excluded those relating to the backbone structure, which are not applicable to non-proteins.

**Figure 2:**
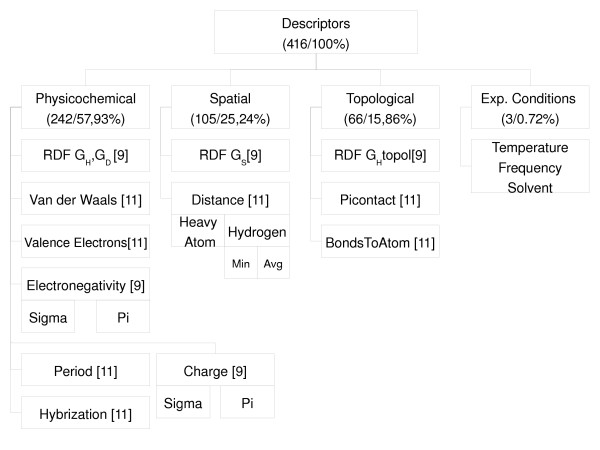
**Hierarchy of the descriptor set, with proportion of types.** Taken from ^1 ^= [9] and ^2 ^= [12].

An overview of the descriptors is shown in figure 2. A more detailed description of the descriptors is shown in table 1 of additional file 1. The calculated descriptors for the whole database are available in  additional file 2.

In order to encode the 3-dimensional environment in the descriptors of a particular atom (and not just the atom itself) Meiler [12] suggests to calculate a set of 10 basic descriptors for the atom the hydrogen is bound to and for the 16 atoms closest by bond (topological) and for the 16 atoms closest in space (geometrical) (if not yet included), thus producing a 3-dimensional basis. Calculating these altogether 330 descriptors (2(16*10)+10) is relatively easy since a small set of descriptors is repeatedly calculated for different atoms. As a notable difference to Meiler's work, which tackles large proteins, in our case the 16 closest atoms by bond often already contain most or all atoms of a small organic molecule.

The set of descriptors suggested by Aires-de-Sousa and coworkers [9] has some descriptors being physico-chemical (charges, electronegativity) and additionally average/minimum/maximum values of atoms in the spheres round the proton are used. The radial distribution function (RDF) of several factors (charge, shielding, electronic current in double bonds, etc.) adds sensitivity for the 3-dimensional environment of the atom in question. RDFs contain informations about the interatomic distances in a molecule, unweighted or weighted by different atomic properties such as atomic mass, electronegativity, van der Waals volume and atomic polarisability [17]. Aires-de-Sousa also suggests to divide the atoms into four groups: protons attached to atoms in aromatic rings, non-aromatic *p *systems, rigid aliphatic systems and non-rigid aliphatic systems.

The descriptors suggested by Meiler [12] were slightly modified by taking first the 16 closest atoms in space and then the 16 closest by bond, if not included in the first set (the other way round than suggested), which we found to give an improved result. From the measurement conditions (temperature, solvent, frequency) only the solvent was kept in the final feature set, however a large percentage of those entries were unknown solvents.

### Test- and training data set

**Figure 3:**
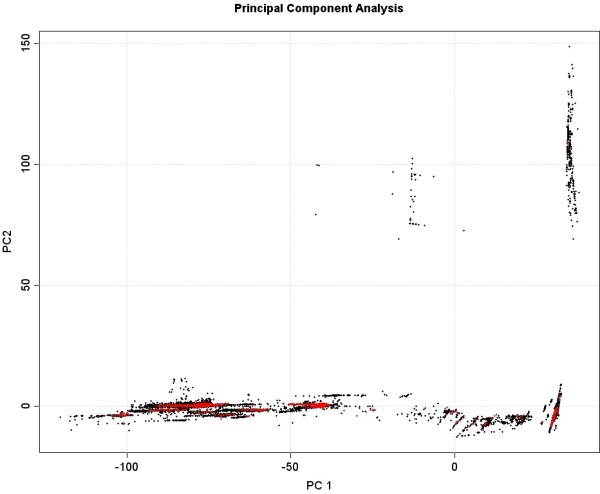
**Coverage of chemical space by NMRShiftDB.** A set of atomic descriptors were calculated for protons in Pubchem (50,000 structures/465,000 protons) (in black) and the protons with assigned shifts in the NMRShiftDB (in red, 1829 structures/18692 protons attached to carbons) and plotted using principal component analysis.

Compounds for training and test data are taken from the NMRShiftDB database, an open access, open submission, open source database for small organic molecules and their assigned NMR spectra [18,19]. The data quality in NMRShiftDB has recently been evaluated in a separate study based on cross validation with data from an external data source [20]. All molecules shown in this paper can be accessed via their molId on  using the "Go directly to ID" function. The assignments in NMRShiftDB are all hand-curated and checked by human reviewers. At the time of writing it comprised 20199 structures with altogether 23722 spectra, of which 2983 were proton spectra (some structures have multiple proton spectra). Successful predictions previously reported were done with much smaller data sets [[9], p. 81]. It should be noted that the NMRShiftDB data have been collected over time by different contributors, they are not in any way selected for the purpose of prediction. To verify a broad coverage of the known organic chemistry space we mapped our data to a descriptor space defined by one of the largest available collections of organic structures, the Pubchem database [21], which we downloaded from . The mapping was done by calculating for each proton a selected set of atomic descriptors such as effective polarisability, *π*/*σ *partial charge and *s *electronegativity for the proton itself, *π*/*σ *electronegativity and partial charge for the connected heavy atom, and similar descriptors applied to spatially neighbouring atoms (see figure 2). The descriptor values from both data sets were analysed by PCA analysis, shown in figure 3. The NMRShiftDB is by far smaller than Pubchem, but the main clusters of the Pubchem data are represented in NMRShiftDB as well.

**Figure 4:**
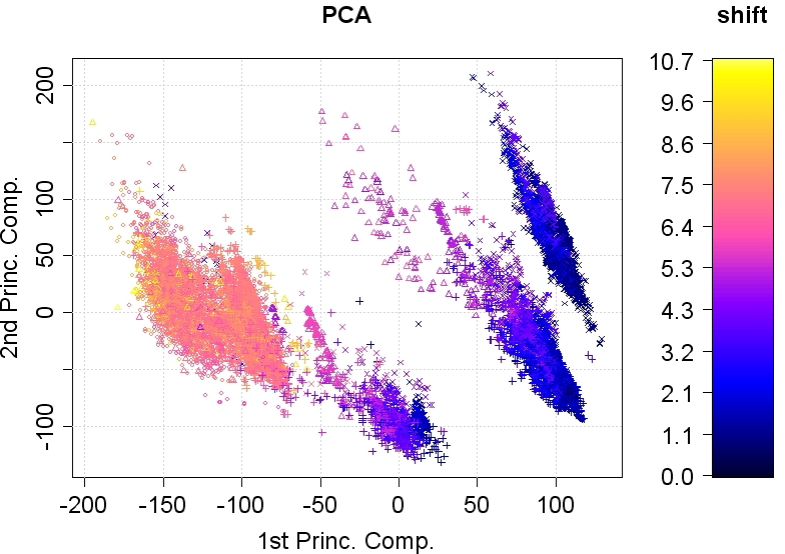
**PCA analysis of the numerical attributes in the descriptor set.** The symbols correspond to the four different proton categories suggested in [9]. "○" for protons in aromatic rings, "△" for protons in non-aromatic *π *systems, "+" for protons in rigid aliphatic systems and "×" for protons in non-rigid aliphatic systems.

For the shift prediction we extracted the protons directly attached to a carbon. Shifts on hetero-atoms are much more influenced by the experimental conditions [[9], p. 82]), which are not subject of our study. The resulting set consists of 18692 measured ^1^H shift values from 0 to 10.7 ppm of 1829 different unique molecules with a mass range of 20 amu to 2000 amu (figure 4).

The protons were categorised as suggested in [9]: a) aromatic rings 21%, b) non-aromatic *π *systems 7%, c) rigid aliphatic systems 27% and d) non-rigid aliphatic systems: 45%.

For each proton we calculated the descriptors described in table 1 of additional file 1. Attributes containing more than 50% missing values (NA) were omitted from the data set. This affected mainly the more distant sample points of the RDF based descriptors and the higher ranks of the 16 topologically closest atoms not contained in the 16 closest by distance. Numerical attributes containing less than 50% NA values were imputed with their mean values. NA values in categorial columns were treated as independent values themselves and were not imputed. After preprocessing the data set was of size 18672 * 246 attributes (207 numerical and 39 nominal). Normalising the data was performed by scaling to the maximum value attribute-wise for numerical attributes. Nominal attributes were assigned integer values in increasing order for distinct attribute levels and scaled accordingly to the same range as the numerical attributes. For clustering and regression purposes, and for computation of distance matrices, we concentrated solely on the numerical attributes. However, in terms of the classification tasks we combined both nominal and numerical attributes.

### Calculation of 3D coordinates

Three dimensional coordinates are often not available to the experimentalist and are not included in NMRShiftDB. They were calculated using the software package CORINA (COoRdINAtes) (Molecular Networks GmbH, Erlangen, Germany [22,23]), the industry standard for coordinate generation. It respects stereo hints (wedge bonds) given in the structures, so that conformations measured and entered by the contributor are taken into account. When a spectrum is measured in solution different conformations usually contribute to the observed chemical shifts. This situation makes the definition of the 3D environment of a proton rather difficult. For the structures in NMRShiftDB, where several conformations are possible a plausible one of them was chosen by CORINA.

### Algorithms for prediction

A large number of algorithms for classification tasks and regression have been proposed in the past. We have used algorithms integrated in the two open source projects R 2.5.1 [24] and Weka 3.5.6 [25] because the consistent programming interfaces allowed to evaluate a broad range of algorithms. As a result of preliminary tests we thoroughly investigated the performance of the following algorithms for the prediction tasks.

#### Instance based learners

Ibk [26] is an instance based learner with k being the number of neighbours. It predicts new data by simply considering the class values of the k nearest neighbours, thereby using a statistical method to 'weed out' irrelevant or noisy instances. It belongs to the class of so called lazy learners, because no concepts are produced, it merely stores the typical values without processing the data in any way.

#### Support Vector Machines

Support vector machines are based on statistical learning theory and belong to the class of kernel based methods [27]. The basic concept of SVMs is the transformation of input vectors into a higher dimensional feature space where a linear separation may be possible between the class members. In this feature space the support vector learning algorithm maximises the distance between the class members of the training set in order to achieve a good generalisation.

#### Decision Trees

Decision Trees recursively split attributes in a top-down manner. The attribute with the highest normalised information gain (based on the concept of entropy used in information theory) is used to make the split decision. The algorithm then recurses down the tree until a leaf with the minimum desired number of instances is reached. A well-known decision-tree algorithm is ID3 and its successor C4.5 [[28], p.115].

#### Random Forest

The random forest classifier was developed by Leo Breiman and Adele Cutler [29] and consists of many decision trees. The algorithm combines Breiman's "bagging" [30] idea and Ho's "random subspace method" [31] to construct a collection of decision trees with controlled variations. A training set for a tree is constructed by choosing N samples with replacement from all N available training cases (bootstrapping). At each node a random subset of variables is used to determine the splitting decision. Finally the mode of all classes by the individual fully grown and unpruned trees is returned.

#### Bagging

Bagging (or Bootstrap and aggregating) was proposed by Leo Breiman in 1994 to improve the classification by combining classifications of randomly generated training sets. Bagging can be used with any type of model. New training sets are constructed by sampling uniformly from the original training set with replacement. The models are fitted using the bootstrap samples and combined by averaging the output (in case of regression) or voting (in case of classification).

#### Boosting

Boosting is a form of reinforcement learning [32]. For each call a distribution of weights is updated to indicate the importance of examples in the data set for the classification. On each round, the weights of each incorrectly classified example are increased, so that the new classifier focuses more on those difficult examples.

#### Multivariate Linear Regression

This is a generalisation of a simple linear regression of two variables. A continuous metric response variable y (i.e. the shift) gets approximated through a linear combination of *n *multiple influence quantities and has the form: *y *= *a*_1 _* *x*_1 _+ ... + *a*_n _* *x*_*n *_+ *b *+ ϵ.

### Feature selection

To reduce the number of input variables without a manual (and error prone) choice, we used the variable importance measure as reported by random forest to select the relevant features associated with the target class.

A random forest constructs *N *unpruned trees by drawing *N *bootstrap samples. The variable importance measurement is performed on the test set established by the bootstrap method: Given a data set of *d *tuples the data set is sampled *d *times with replacement constituting the training set. The data tuples that did not make it into the training set form the test set. The probability of not being chosen is (1 - 1/*d*)^*d*^. If *d *is large, the probability approaches *e*^-1 ^= 0.368. Thus, 63.2% form the training set and 36.8% form the test set. The random forest procedure provides two importance measures:

• Mean Decrease Accuracy (%IncMSE): It is constructed by permuting the values of each variable of the test set, recording the prediction and comparing it with the unpermuted test set prediction of the variable (normalised by the standard error). For classification, it is the increase in the percentage of times a test set tuple is misclassified when the variable is permuted. For regression, it is the average increase in squared residuals of the test set when the variable is permuted. A higher %IncMSE value represents a higher variable importance.

• Mean Decrease Gini (IncNodePurity): Measures the quality (NodePurity) of a split for every variable (node) of a tree by means of the Gini Index. Every time a split of a node is made on a variable the gini impurity criterion for the two descendent nodes is less than the parent node. Adding up the gini decreases for each individual variable over all trees in the forest gives a fast variable importance that is often very consistent with the permutation importance measure. A higher IncNodePurity value represents a higher variable importance, i.e. nodes are much 'purer'.

The two variable importance measures are drawn separately for each tree of the forest and finally are averaged over all trees of the forest and there is no clear guidance on which measure to prefer.

## Results

Before applying the machine learning methods, we examined the characteristics of the molecular descriptors in our evaluation data set.

### Numerical attributes

**Figure 5:**
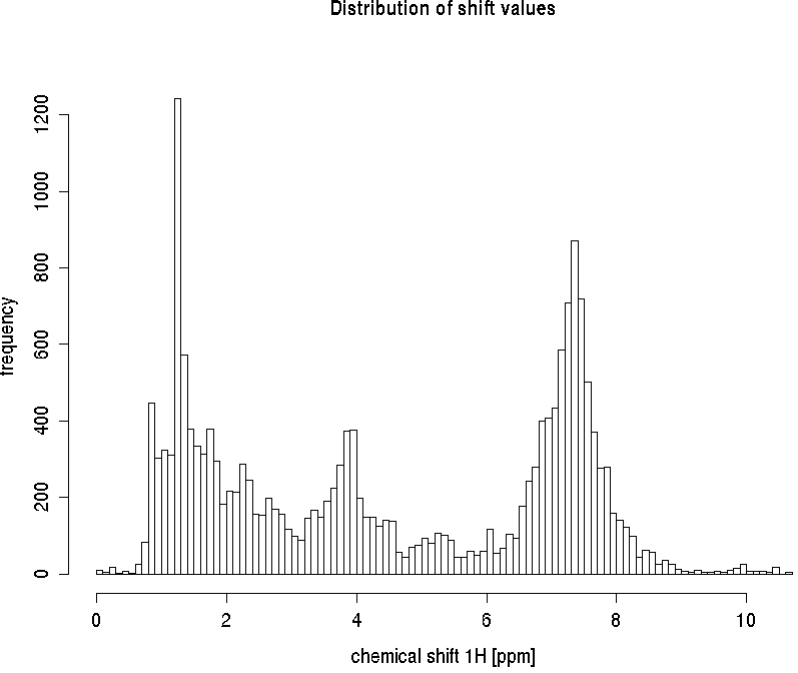
**Histogram of the shift values.** Most prominent regions are aromatic and and aliphatic values at 1.3 and 7.4 ppm respectively.

We performed a PCA analysis of the 207 numerical attributes. In figure 5 four major groups are visible. The bulky group on the left side consists in fact of two subgroups, predominantly representing aromatic rings including a heteroatom or protons contained in aliphatic ring systems with adjacent *p *systems. The group in the lower center contains e.g. aliphatic protons with adjacent oxygens (aldehyde, etc.), whereas at the center to the right there are mostly aliphatic ring systems with heteroatoms and on the far right side predominantly alkyl-groups are visible. Broadly speaking there is a strict linear separability achievable by drawing a line from the top left-hand corner to the bottom right-hand corner of the diagram. Even the two groups on the left side are strictly linear separable. Each proton in the diagram carries a symbol according to its proton category and is coloured by its shift value. There is a general trend for decreasing shift values from left to right, but within groups variability is rather strong. Notably, the four different proton categories do not unambiguously reflect the visibly detectable groups. The corresponding loadings plot is provided in figure 1 of additional file 1, and reveals that especially the (spatially) nearest heavy atoms influence the separation into the groups shown.

### Categorial attributes

**Figure 6:**
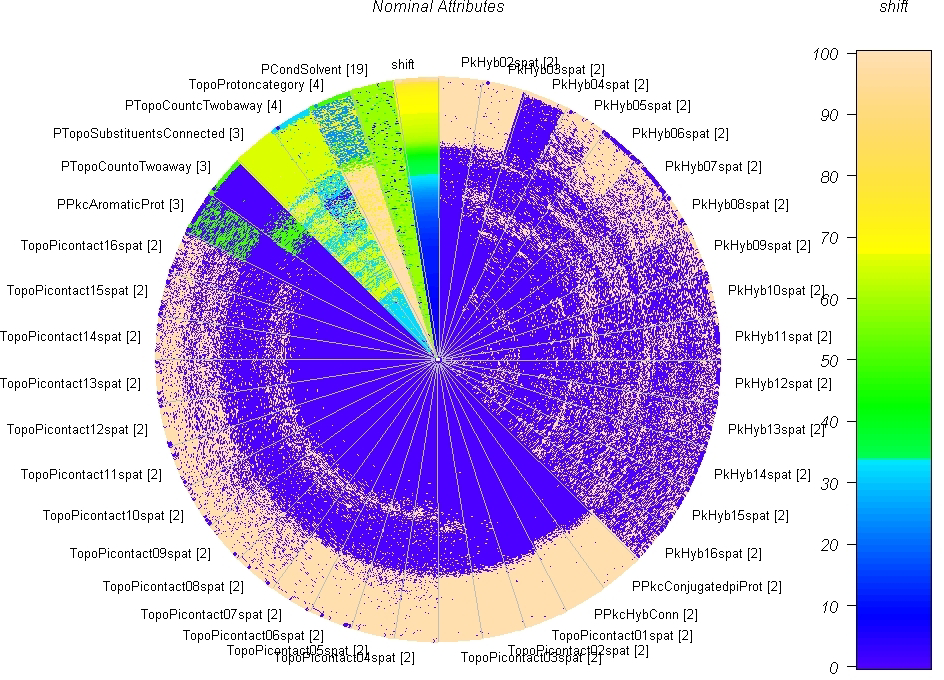
**Circle Segments Plot of the categorial attributes in the descriptor set.** The values in each of the segments are sorted increasingly according to the shift from the inner of the circle to the outer. The chemical shift in the range [0..10.7] is mapped to the coloured interval of size [0..100]. The number of the distinct attribute levels *i *is given in square brackets. Those values are mapped to the colour map as *i*/max(*i*) * 100. The influence of the dichotomous spatial variables PkHyb* and TopoPicontact* are less amenable with respect to the chemical shift, the farther the respective heavy atom is.

Figure 6 shows a circle segments plot of the 39 nominal attributes. The descriptors PkHybXspat and TopoPiContactXspat are two descriptors (hybridization and so-called *p *contact [[12], p. 28]) calculated for the atoms around the proton in increasing distance. It confirms common NMR knowledge that the influence on the shift is less prominent the further away an atom is. The circle segments plot shows that with less homogenous distributed attribute levels. Within the PkHybXspat hybridisation there is a strong decline of the influence from the fourth neighbour. We are not aware of any physicochemical effect which can explain this behaviour.

### Machine learning comparison

**Figure 7:**
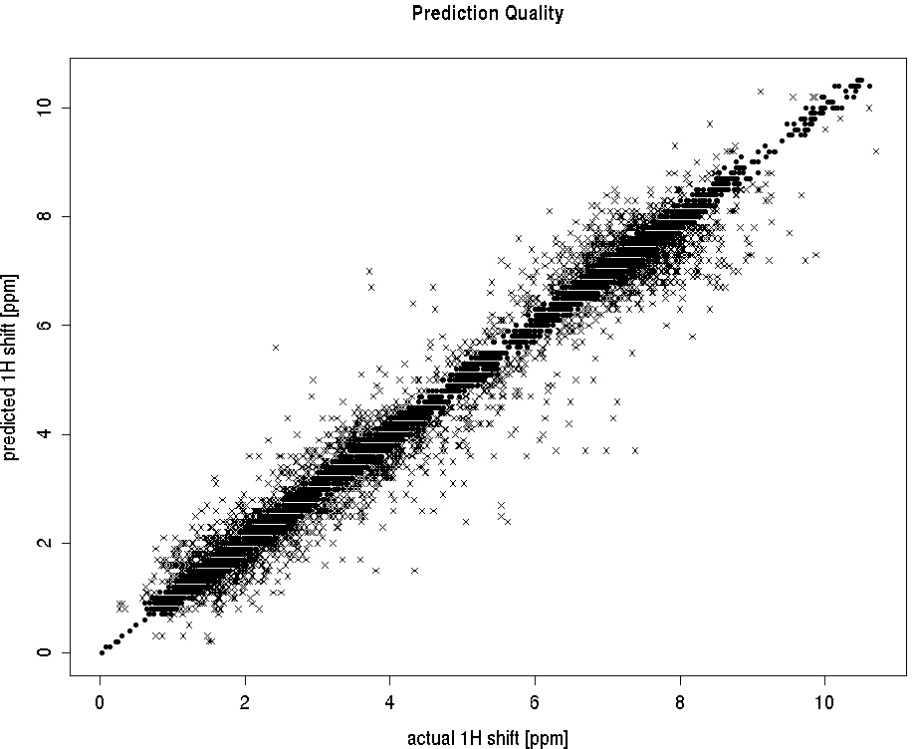
Comparison of the real shift values and the predicted values on the data set (18672 protons) for a Random Forest with selected features.

We used the data set presented in the last section to evaluate the performance of the selected machine learning methods and training strategies. The most simple approach is the Multivariate Linear Regression based on the numeric descriptors alone, which has an MAE of 0.29 ppm. Using the same descriptor set, but the non-linear SVM regression, the result can be improved to 0.21 ppm. The J48 and Random Forests use the categorial descriptors in addition to the numeric features. An illustrative comparison of the actual versus the predicted shift values using Random Forest can be found in figure 7.

The settings for the algorithms were mostly according to default settings provided by the packages used in this study. Best results for J48 were obtained for a minimum number of two instances per leaf. A larger number of instances deteriorated the performance slightly. For the Random Forest we used an unlimited depth of trees and the default number of trees (10). For the instance based learners (IBk) the optimal number of neighbours was one, larger number of nearest neighbours again worsen the performance gradually. For the support vector regression we utilised the radial basis kernel and a grid search for the optimal parametrisation values.

Boosting had the largest impact on the J48 classifier, both when used alone and in combination with feature selection. We also evaluated the Bagging (or Bootstrap aggregating) training method, and achieved a marginally larger error compared to boosting (data not shown).

**Figure 8:**
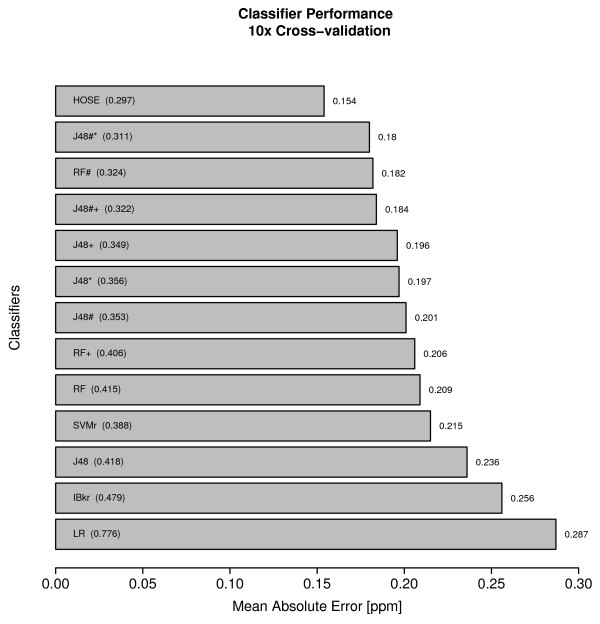
**Mean absolute error of investigated Classifiers, the standard error (SE) is given in brackets.** Classifiers trained with selected features are annotated with an additional #. Bagged Classifiers are annotated with an additional * and boosted classifiers carry an additional +- symbol. MAE/SE calculated with two decimal place for *δ*_*exp *_and one decimal place for *δ*_*pred *_in terms of classification.

The accuracy rate as given by the mean absolute error (ten fold cross validated) of the prediction algorithms is shown in figure 8. The mean absolute error varies between 0.15 ppm and 0.29 ppm depending on the method. Linear Regression, being a simple method, performs worst (but still delivers reasonable results).

**Figure 9:**
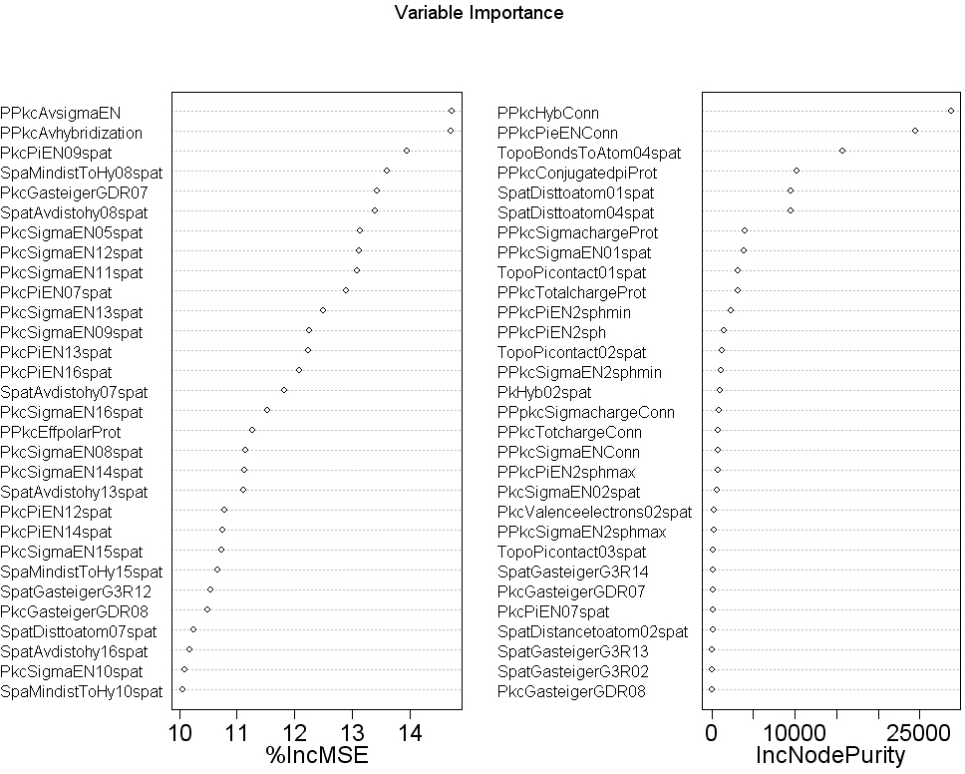
**Mean Decrease Accuracy (%IncMSE) and Mean Decrease Gini (IncNodePurity) (sorted decreasingly from top to bottom) of attributes as assigned by the random forest.** The abbreviations of the descriptors can be found in table 1 of additional file 1. For the mean decrease in accuracy the most relevant descriptors either relate to the proton or the carbon it is connected to or to atoms close to this. The most important types of descriptors are hybridisation, electronegativity, distances and whether the proton is joined to a conjugated *π *system.

Figure 9 shows the first 30 features ordered by two specific importance measures. For the training we have used the best 25 descriptors from the the Mean Decrease in Accuracy. These cover both numeric and categorial attributes, so we applied them to RF and J48 only.

### Misclassifications

**Figure 10:**
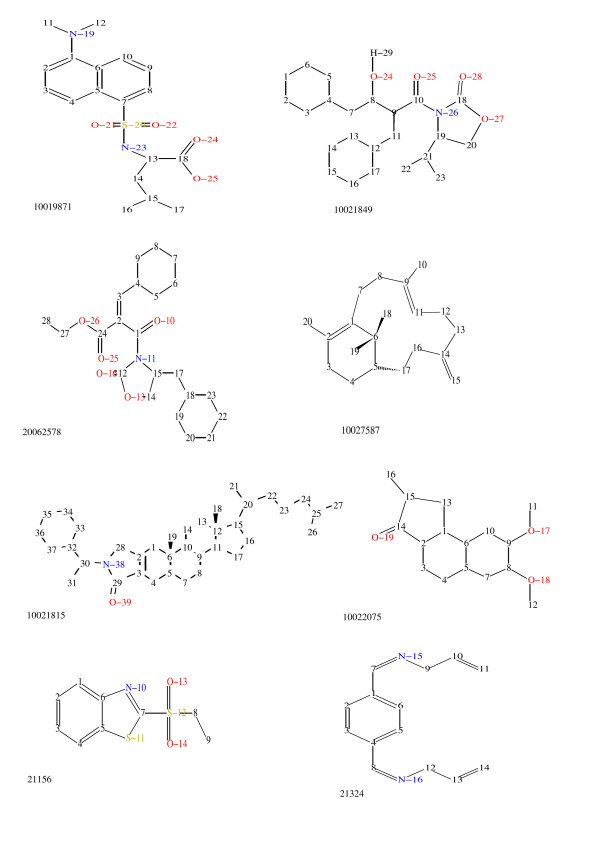
**Representative examples for typical misclassifications ('hard errors').** NMRShiftDB-molid: 10019871, atomID: 10189925 at heavy atom nr. 16 (0.62 ppm), atomID: 10189928 at heavy atom nr. 17 (0.27 ppm). NMRShiftDB-molid: 10021849, atomID: 10324987 at heavy atom nr. 22 (0.73 ppm), atomID: 10324990 at heavy atom nr. 23 (0.27 ppm). NMRShiftDB-molid: 20062578, atomID: 20958256 at heavy atom nr. 17 (3.53 ppm), atomID: 20958257 at heavy atom nr. 17 (2.72 ppm). NMRShiftDB-molid: 10027587, atomID: 11110950 at heavy atom nr. 7 (2.11 ppm), atomID: 11110951 at heavy atom nr. 7 (1.45 ppm). NMRShiftDB-molid: 10021815, atomID: 10323000 at heavy atom nr. 13 (1.96 ppm), atomID: 10323001 at heavy atom nr. 13 (1.11 ppm). NMRShiftDB-molid: 10022075, atomID: 10335470 at heavy atom nr. 13 (3.61 ppm), atomID: 10335471 at heavy atom nr. 13 (2.90 ppm). NMRShiftDB-molid: 21156, atomID: 283794 at heavy atom nr. 1 (8.21 ppm). NMRShiftDB-molid: 21324, atomID: 296084 at heavy atom nr. 2 (6.73 ppm). Provided shift values are experimental and not predicted shifts. Predictions are given in table 2. The corresponding descriptor set is provided in table 2 of additional file 1. The HOSE codes of these atoms are shown in table 4. The  different descriptor sets for atoms 10189925 and 10189928 are shown in  table 3.

Diastereotopic protons attached to carbon atoms are topologically equivalent but face different 3D environments due to a single 3D conformation being more or less randomly chosen for our computation. As has been stated before, the use of a single 3D conformation is artificial and introduces a potential source of error. A rigorous treatment would create a set of the most frequent 3D conformation in solution as well as an assessment of their likelihood of occurrence. Shifts would need to be predicted for each conformation and then averaged weighted by their probability. This approach would a) generate computation times rendering the application useless for our purpose and b) require training data with measured shifts for single conformations in solution – something which is clearly not achievable but could be approximated through rigorous ab-initio calculations of chemical shifts for ensembles of conformations. For many methylene proton pairs and any dimethyl group that is a rotatable fragment, accounting for multiple conformations would average out differences such as those seen figure 10 (Tables 3 and 4 provide further data for this, see legend).

**Table 3:** The set of descriptors with distinctive values for the atomIds of CH3 protons from NMRshiftDB-molid: 10019871 at heavy atom 16 and 17, from figure 10 (topleft).

	10189925	10189928
PkcPeriod12spat	20.00	40.00
PkcPeriod13spat	40.00	20.00
PkcPeriod15spat	20.00	40.00
PkcPiEN16spat	16.57	0.00
PkcSigmaEN12spat	73.73	79.00
PkcSigmaEN13spat	78.73	73.60
PkcSigmaEN15spat	80.59	97.03
PkcValenceelectrons12spat	14.29	57.14
PkcValenceelectrons13spat	57.14	14.29
PkcValenceelectrons15spat	14.29	85.71
PkcVdwradius12spat	57.14	80.95
PkcVdwradius13spat	80.95	57.14
PkcVdwradius15spat	57.14	72.38
PkHyb15spat	0.00	100.00
SpaMindistToHy08spat	38.80	29.55
SpaMindistToHy12spat	42.07	29.40
SpaMindistToHy14spat	42.40	36.07
SpaMindistToHy15spat	37.96	25.46
SpaMindistToHy16spat	39.37	29.20
SpatAvdistohy12spat	44.16	37.44
SpatAvdistohy14spat	44.58	37.87
SpatAvdistohy15spat	43.27	34.91
SpatAvdistohy16spat	42.74	36.12
SpatDisttoatom12spat	42.70	36.98
SpatDisttoatom14spat	44.74	37.29
SpatDisttoatom15spat	43.94	37.62
SpatDisttoatom16spat	41.95	35.81
TopoBondsToAtom08spat	15.79	21.05

**Table 4:** HOSE Codes for given atomIDs (hard errors)

atomIds	Hose Code
10189925	H-1;C(HHC/HCC/HHC,HHH)HCN/=OO,HS/
10189928	H-1;C(HHC/HCC/HHC,HHH)HCN/=OO,HS/
10324987	H-1;C(HHC/HCC/HCN,HHH)HHO,CC/&,=OC,=O&/
10324990	H-1;C(HHC/HCC/HCN,HHH)HHO,CC/&,=OC,=O&/
20958256	H-1;C(HCC/HCN,*C*C/HHO,CC,H,H,*C,*C)&,=OC,=O&,H,H,*C,*&/,,=CC,,H*&/
20958257	H-1;C(HCC/HCN,*C*C/HHO,CC,H,H,*C,*C)&,=OC,=O&,H,H,*C,*&/,,=CC,,H*&/
11110950	H-1;C(HCC/=CC,HHC/CC,CCC,=CC)HHC,HHH,HC&,HHH,HHH,HC,HHH/HH&,HHC,HHC/
11110951	H-1;C(HCC/=CC,HHC/CC,CCC,=CC)HHC,HHH,HC&,HHH,HHH,HC,HHH/HH&,HHC,HHC/
10323000	H-1;C(HCC/CCC,HHC/H,H,C,C,C,C,HHH,HC&)H,H&,C,C,C,HH,HH&,&,CCC/,HH,HH,C,C,HHH,H&C,HHC,HHH/
10323001	H-1;C(HCC/CCC,HHC/H,H,C,C,C,C,HHH,HC&)H,H&,C,C,C,HH,HH&,&,CCC/,HH,HH,C,C,HHH,H&C,HHC,HHH/
10335470	H-1;C(HCC/HCC,*C*C/=O&,HHH,*C,*C,*C,&),*C*C,H,H*C,*&/,H,H*C,*&,*&O/
10335471	H-1;C(HCC/HCC,*C*C/=O&,HHH,*C,*C,*C,&),*C*C,H,H*C,*&/,H,H*C,*&,*&O/
283794	H-1;C(*C*C/*C*N,H*C/*C*S,*C,H*&)H*&,*&,*&S/,=O=OC/
296084	H-1;C(*C*C/*CC,H*C/H*C,H=N,*&C)H*&,C,H=N/,HHC,C/

**Figure 11:**
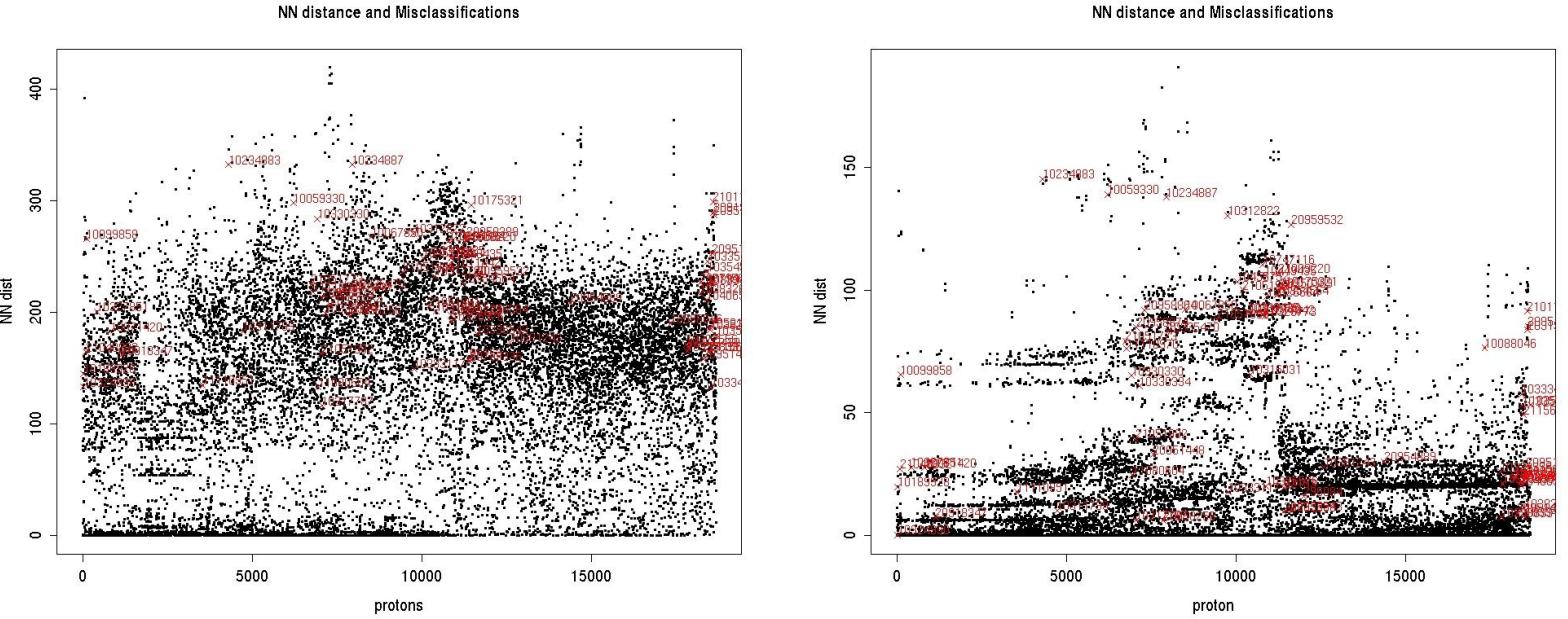
**Effect of feature selection on quality of prediction.** Protons in black are those correctly classified by at least one classifier, the misclassified protons ('hard errors') are shown in red with the corresponding atomID. Left: unreduced descriptor set. Right: reduced descriptor set after feature selection. The protons are sorted increasingly according to the shift value [0..10.7] ppm. On the y-axis the Manhattan distance to the nearest neighbour (NN) proton in the numeric descriptor space is shown.

There are a number of protons for which all of the investigated machine prediction algorithms (except HOSE Codes) at a time fail to correctly predict the chemical shift with an underlying error rate of > 0.5 ppm (which we call 'hard errors'). The shifts of these protons cannot accurately be predicted with either a reduced or the full descriptor set or any special settings for the algorithms. Therefore they are affected by a fundamental problem. In figure 11 the 'hard-error' – misclassifications contributing to the overall error are displayed. From this plot one can judge, that the 'hard-error' – misclassifications do not arise because they are outliers. When scrutinising those erroneous shift predictions in detail we encountered the following types of misclassifications (examples mentioned are shown in figure 10):

• Diastereotopic methyl-groups (moldID: 10019871 and 10021849).

• Methylene-protons in chains (moldID: 20062578 and 10027587).

• Methylene-protons in rigid rings (molID: 10021815 and 10022075).

• False entries such as (molID: 21156 and 21324). Here virtually no systematic errors were detected.

In addition, training data may be imperfect due to missing stereochemical information for diastereotopic protons. If no stereochemistry is given in NMRShiftDB, the shifts are randomly assigned to the atoms in space. If this assignment is wrong and there are enough correct examples in the database, the prediction may still be right, but different from the actual assignment. An example for this are most likely atomIDs 10335470 and 10335471 in Table 2. We believe that those types of false assignments may be encountered in the database to a not negligible amount. Consequently, when applying feature selection a potential set of descriptors, possibly explaining the difference in shifts, are clipped away, because due to the false assignments they become meaningless. Note that in figure 11 for the indices between approximately 12600 and 17200 there is only one single ('hard error') – misclassification. This corresponds to the shift range of 6.9 ppm to 7.7 ppm, where most of the protons are attached to aromatic six membered rings.

**Table 2:** Predictions of hard errors for all classifiers in ppm.

atomID	shift	LR	HOSE	J48	J48+	J48*	J48#+	J48#*	J48#	RF	RF#	RF+	IBk	SVM
10189925	0.62	0.91	0.85	0.90	0.80	0.90	0.90	0.90	0.90	0.90	0.90	0.90	0.87	0.91
10189928	0.27	0.94	0.85	0.80	0.90	0.90	0.90	0.90	0.90	0.80	0.90	0.90	0.78	0.94
10324987	0.73	1.05	0.95	0.80	0.80	0.80	0.80	1.10	0.80	0.80	0.80	0.80	0.82	0.87
10324990	0.27	1.16	0.95	0.90	0.90	1.10	1.00	0.80	0.80	1.10	0.80	1.10	1.33	0.79
20958256	3.53	2.43	3.01	2.70	2.70	2.70	2.70	2.70	2.70	2.70	2.70	2.70	2.72	2.82
20958257	2.72	2.43	3.01	2.70	3.50	3.50	3.50	3.50	3.50	3.50	3.50	3.50	3.53	2.91
11110950	2.11	1.68	2.24	1.40	1.40	1.40	2.30	2.30	1.40	2.00	1.40	1.40	1.45	2.24
11110951	1.45	1.96	2.24	2.10	2.10	2.10	2.30	2.30	2.10	2.00	2.10	2.10	2.11	2.33
10323000	1.96	1.79	1.52	1.40	1.10	1.10	1.10	1.10	1.10	1.10	1.10	1.10	1.11	1.45
10323001	1.11	1.82	1.52	1.40	2.00	2.00	2.00	2.00	1.40	2.00	2.00	2.00	1.96	1.48
10335470	3.61	2.92	2.91	2.90	2.90	2.90	2.90	2.80	2.90	2.90	2.90	2.90	2.90	3.06
10335471	2.90	2.87	2.91	2.90	3.60	3.60	3.60	3.60	3.60	3.60	3.60	3.60	3.61	3.08
283794	8.21	7.28	8.04	7.00	7.50	7.00	7.20	7.20	6.70	7.60	8.00	7.50	7.47	7.29
296084	6.73	7.33	7.52	7.70	7.50	7.40	8.10	7.60	7.60	8.10	7.60	7.40	7.38	7.53

### HOSE codes

**Figure 12:**
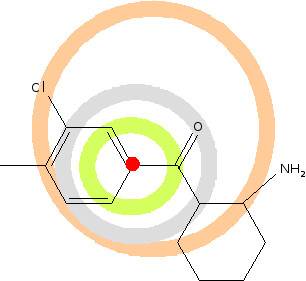
**Principle of HOSE codes.** The HOSE code is built sphere-wise around the atom described; the carbon shown would have the HOSE code (four spheres): C-arom;*C*CC(*C,*C,=OC/*CX,*&,,CC/&C,,CN,C).

To compare the machine learning algorithms with a fundamentally different approach, we used HOSE (Hierarchically Ordered Spherical description of Environment) codes for the prediction (figure 12). Table 5 illustrates the connection of HOSE codes and chemical shifts. HOSE codes [5] are a well established method for prediction of especially ^13^C-NMR spectra. Their use for ^1^H spectra is discouraged since they do not include three-dimensional information. However, we wanted to see how they performed and did a cross-validation with HOSE codes on the same data set as used for the other methods with a six sphere fall back. This means that we created a six-sphere HOSE code for each atom in the test set and tried to match this HOSE code in the "training" set (a real training process is obviously not involved). If one or more values were found the average was considered for prediction. If no matches were found, we backed up sphere by sphere until a hit was found. In the worst case it will be found in sphere one, since that contains the atom neighbour and the bond order, which for protons is always a single-bonded carbon (since we only predict protons on carbons). Alternatively, an averaging of the lexicographically next neighbors in the next sphere could be employed. To our surprise, HOSE codes produced the lowest mean absolute error of 0.154 of all methods tested (figure 8).

**Table 5:** A lexicographically ordered section of NMRShiftDB's table of HOSE codes and assiciated shifts to illustrate the connection between similar HOSE codes and similar NMR shifts.

Hose Code	Shift
H-1;C(*C*C/*CC,*CO/*CO,CCC,*&C,H)H*&,H,HHH,HHH,HHH,CCC/,HHH,HHH,HHH/	6.89
H-1;C(*C*C/*CC,*CO/*CO,H=C,*&C,C)H*&,C,CC,H=C,HHC/,HHC,*C*C,%N,CC,HHC/	7.88
H-1;C(*C*C/*CC,*CO/*CO,H=C,*&C,C)H*&,C,CC,H=C,HHC/,HHC,*C*C,%N,HC,HHC/	7.88
H-1;C(*C*C/*CC,*CO/*CO,H=C,*&C,C)H*&,C,HC,CCC,HHC/,HHC,*C*C,HHH,HHH,HHH,HHC/	7.19
H-1;C(*C*C/*CC,*CO/*CO,H=C,*&C,C)H*&,C,HC,H=C,=OC/,=OC,*C*N,HC,,HHH/	7.18
H-1;C(*C*C/*CC,*CO/*CO,H=C,*&C,C)H*&,C,HC,H=C,HHC/,HHC,*C*C,CC,HHC/	7.14
H-1;C(*C*C/*CC,*CO/*CO,H=C,*&C,C)H*&,C,HC,H=C,HHC/,HHC,*C*C,HC,HCC/	7.14
H-1;C(*C*C/*CC,*CO/*CO,H=C,*&C,C)H*&,C,HC,H=C,HHC/,HHC,*C*C,HC,HHC/	7.13
H-1;C(*C*C/*CC,*CO/*CO,H=C,*&C,C)H*&,C,HC,H=C,HHC/,HHC,*N*N,HC,HHC/	7.13
H-1;C(*C*C/*CC,*CO/*CO,H=C,*&C,C)H*&,C,HC,H=C,HHC/,HHC,=OO,HC,HHC/	6.99
H-1;C(*C*C/*CC,*CO/*CO,H=C,*&C,C)H*&,C,HC,H=C,HHH/,HHH,*C*C,HC/	7.10
H-1;C(*C*C/*CC,*CO/*CO,H=C,*&C,C)H*&,C,HC,H=O,HHC/,HHC,*C*C,,HCC/	7.20
H-1;C(*C*C/*CC,*CO/*CO,H=C,*&C,C)H*&,C,HC,H=O,HHC/,HHC,*C*C,,HHC/	7.16
H-1;C(*C*C/*CC,*CO/*CO,H=C,*&C,C)H*&,C,HC,H=O,HHC/,HHC,=OC,,HHC/	7.35
H-1;C(*C*C/*CC,*CO/*CO,H=C,*&C,C)H*&,C,HC,HHH,HHC/,HHC,*C*C,HCC/	7.19
H-1;C(*C*C/*CC,*CO/*CO,H=C,*&Y,C)H*&,C,HC,,HHC/,HHC,*C*C,HHC/	6.89
H-1;C(*C*C/*CC,*CO/*CO,H=C,H*&,C)H*&,C,CC,HHC/,HHC,*C*C,%N,HHC/	7.10
H-1;C(*C*C/*CC,*CO/*CO,H=C,H*&,C)H*&,C,CC,HHH/,HHH,*C*C,%N/	7.20
H-1;C(*C*C/*CC,*CO/*CO,H=C,H*&,C)H*&,C,HC,HHC/,HHC,*C*C,HHC/	7.13

## Conclusion

We have applied a selection of machine learning algorithms to one of the largest public available data sets of ^1^H NMR spectra with the goal of predicting spectra for the use in Computer-Assisted Structure Elucidation of biological metabolites. This result will enable the implementation of open tools for the elucidation of unknown compounds with high-throughput NMR analysis of biological systems.

The best predictions were obtained with HOSE codes, Random Forests, J48 decision trees for mixed categorial/numerical features and Support Vector Machine regression if only the numerical features were used. More important than the choice of the machine learning algorithm is the selection of the molecular descriptors. We were able to drastically improve the overall accuracy by reduction of the full descriptor set from [9] and [12] to the first 25 descriptors shown in figure 9. Instead of a manual (and potentially biased) selection scheme we used the most predictive descriptors reported by the Random Forest. With less input variables the machine learning methods create more compact models and are less prone to over-fitting problems. Furthermore, we found that Bagging and Boosting increased the prediction rate throughout all the predictors for both the regression and classification tasks. We discussed different sources of errors which are mostly related to stereochemical properties of the protons and possible mis-assignments in the data set. In this respect machine learning methods help to discover inconsistencies in large databases to improve the overall data quality.

HOSE codes leading to the best prediction results certainly came as a surprise for proton spectrum prediction because common wisdom expects a significant dependence of proton spectra on 3D effects which are not explicitly taken into account. A downside of HOSE code might be the reliance on a lookup table which requires a large sample set for meaningful predictions. Strictly speaking, HOSE codes cannot be considered as being a predictive classifier, but more rather an elaborated 'look-up-procedure', comparable to the instance based lazy learners like IBk. Compared to HOSE codes, the prominent advantage of the machine learning methods is certainly that they revert to an mathematical model as the underlying predictive basis. Also, three dimensional molecular properties are involved in the prediction.

ACD labs  report a standard error of 0.22 ppm for their software ACD/HNMR 8.0 [33], and CambridgeSoft ChemDraw 8.0 reports 0.45 ppm, while we achieve an intermediate result of 0.31 ppm for the best classifier. Both are computed on a database size of approximately 50,000 shift values, which is not available to the public. A direct quantitative comparison of our results and the commercial software is not possible since (i) size and quality of the data sets differ and (ii) the results are obtained with different evaluation methods (e.g. ACD used a leave-one-out test, while we used a 10 fold cross validation, resulting in a distinct bias-variance ratio). Like any other more or less randomly collected dataset, the NMRShiftDB will face a certain amount of duplication of certain frequently occuring cases. These cases have an increased likelyhood of occuring unchanged in both training and prediction set and may therefore bias the results of especially the HOSE code prediction. This bias is reduced if the number of spheres taken into account increases.

The results we have presented were obtained using straight forward molecular descriptors and proven machine learning methods. For the given data set they can be considered as a baseline system, against which future shift prediction systems can be compared.

## Authors' contributions

SK was lead developer of the NMRShiftDB database and performed the work on descriptor calculation. BE and SN performed the data mining experiments and provided expertise on artificial intelligence. CS conceived and supervised the research project. All authors contributed to the manuscript.

## Supplementary Material

Additional file 1 Supplement. Plots and Tables referenced by main article. Click here for file

Additional file 2 Descriptor set. The data provides the complete set of descriptor values in matrix form for the H-NMR shifts upon the prediction is based. Click here for file
