# Supplementary material for: Building blocks for automated elucidation of metabolites: Machine learning methods for NMR prediction
Source: BMC Bioinformatics. 2008 Sep 25;9:400. doi: 10.1186/1471-2105-9-400 (PMC2605476; doi:10.1186/1471-2105-9-400)
Supplement: Additional file 1 — Supplement. Plots and Tables referenced by main article. [file 1471-2105-9-400-S1.pdf]

Building blocks for automated elucidation of metabolites: Machine learning methods for NMR prediction. Supplemental Material

Stefan Kuhn, Björn Egert, Steffen Neumann and Christoph Steinbeck

August 25, 2008

Supplementary Material

These are additional figures and tables related to our dataset. The additional file `input.csv.gz` includes the input data as comma separated matrix, which can be read by most spreadsheet applications or the R code `data <- read.csv("input.csv", row.names=1)`. The first row is the atomID as stored in NMRshiftDB, the remaining 246 columns are the descriptor values with at most 50% NA values. The categorial descriptors were translated into discrete numeric values, and each descriptor was scaled up to 100.

Descriptors and Data

In this section we provide a more detailed listing of the descriptors used, including the reference to the literature where they have been described (see table 1).

Group	Atom Used	Propery calculated	Abbreviation	Reference
1	Proton	Conjugated π	PPkcConjugatedpiProt	CDK
		Effective Polarizability	PPkcEffPolarProt	
		σ Partial Charge	PPkcSigmachargeProt	[1]
		Total Charge	PPkcTotalchargeProt	
		Protoncategory		
2	Connected	Hybridization	PPkcHybConn	[2]
	Atom	Aromaticity		CDK
		π / σ Partial Charge	PPkc[Pi|Sigma]ChargeConn	[1]
		Total Charge	PPkcTotalchargeConn	
		π / σ Electronegativity	PPkc[Pi|Sigma]ENConn	[1, 2]
		Number Substituents		[2]
3	The 16	π Electro-Negativity	PkcPiENXspat	[2]
	spatially	σ Electro-Negativity	PkcSigmaENXspat	
	closest atoms	Van der Waals Radius		
	(closest to	Hybridisation	PkcHybXSpat	
	connected			
	atom), ordered	Period		
	by distance	Number Valence Electrons	PkcValenceelectrons02spat	
		π-contact	TopoPicontactXspat	
		Bonds to atom in group 2	TopoBondsToAtomXspat	
		Distance to atom in group 2	SpatDisttiatom07spat	
		Min / Avg Distance to Hydrogen(s)	Spat[Av|Min]disttoHyXSpat	
		connected to atom in group 2	(X is the position count)	
4	The 16	Same properties as in group 3		[2]
	topologically			
	closest (to			
	connected atom)			
	atoms (if not in			
	group 3), ordered			
	by bond distance,			
	within distances			
	randomly			
5	All Atoms	Average Hybridisation	PPkcAvhybridization	[2]
	from group 3	Average σ / π Electronegativity	PPkcAv[sigma|pi]EN	
	and group 4	Average number valence electrons		
		Average Reciprocal Distance		
6	All Atoms in	Max/Avg. π Partial Charge		[1]
	second sphere	Max/Min/Avg of π Electronegativity	PPkcPiEN2sph[max|min]	
		Max/Min/Avg of σ Electronegativity	PPkcSigmaEN2sph[max|min]	
		Max/Min/Avg of σ Partial Charge		
		Max/Min/Avg of Total Charge		
		Count of Carbons/Oxygens		
7	Radial Distri-	$g_h(lr)$ (a,c)/ $g_D(lr)(a,c)$	PkcGasteiger[GHR|GDR]X	[1]
	bution Functions	$g_S(lr)$ (a,c)/ $g_3(lr)(c)$	SpatGasteiger[GSR|G3R]X	
	(used for categories		(X is the sampling point)	
	described in "Test-			
	and Training data			
	set")			
8	Specific for non-	σ / π / total charge		[1]
	aromatic π protons,	σ / π electronegativity		
	for all other atoms	Effective polarizability		
	around the double	Conjugated π		
	bond			

Table 1: Table of all descriptors used for prediction.

A simple example for the descriptors is e.g. the predicate "aromaticity", which evaluates whether this atom is in an aromatic system or not. A more complex example is 'π-contact' between two atoms, which determines if there exists a conjugated π-system which contains either both atoms or directly linked neighbors of the atoms [2]. This value is therefore true (or 1), if the atom is in π-contact with the atom in focus and false (zero) otherwise. A numeric descriptor is e.g. the set of 3D Radial Basis Functions (RDFs), which were calculated from the 3D model generated by CORINA. The function $RDF(lr)$ was sampled at a number of points: $RDF(lr) = \Sigma_i \Sigma_j a_i a_j e^{-b(r-r_{ij})^2}$. In this equation, a_i and a_j are properties of atoms i and j, $r_i j$ is the 3D distance between the atoms i and j, and b is a smoothing parameter. This is a function telling how many atoms are probably found at a certain distance around an atom. It is a probability function since most bonds are not fixed and atoms move. The values of this continuous function at certain distances are used as descriptors with a distance weighting $P = \frac{1}{N*R}\Sigma_{i=1}^{N} p_i r_i^{-1}$.

Hard Errors

Table 2 shows the descriptors as calculated for the atoms which cause hard errors. See section "Misclassifications" of the paper for details.

	10189925	10189928	10324987	10324990	20958256	20958257	11110950	11110951
PCondSolvent	59.38	59.38	59.38	59.38	59.38	59.38	64.06	64.06
PkcGasteigerGDR00	65.59	65.59	65.59	65.59	65.59	65.59	66.29	66.34
PkcGasteigerGDR01	73.80	73.80	73.80	73.80	73.80	73.80	77.50	77.51
PkcGasteigerGDR02	64.92	64.92	64.92	64.92	64.92	64.92	69.16	69.20
PkcGasteigerGDR03	88.68	88.68	88.68	88.68	88.68	88.68	88.73	88.80
PkcGasteigerGDR04	89.50	89.50	89.50	89.50	89.50	89.50	88.92	88.96
PkcGasteigerGDR05	89.37	89.37	89.37	89.37	89.37	89.37	88.80	88.67
PkcGasteigerGDR06	95.33	95.33	95.33	95.33	95.33	95.33	95.33	94.86
PkcGasteigerGDR07	87.56	87.56	87.56	87.56	87.56	87.56	88.34	87.08
PkcGasteigerGDR08	52.66	52.66	52.66	52.66	52.66	52.66	52.98	52.69
PkcGasteigerGDR09	28.56	28.56	28.56	28.56	28.56	28.56	28.48	28.50
PkcGasteigerGDR10	15.90	15.90	15.90	15.90	15.90	15.90	15.32	15.52
PkcGasteigerGDR11	31.36	31.36	31.36	31.36	31.36	31.36	30.48	30.88
PkcGasteigerGDR12	32.10	32.10	32.10	32.10	32.10	32.10	31.90	31.88
PkcGasteigerGDR13	50.05	50.05	50.05	50.05	50.05	50.05	50.17	50.05
PkcGasteigerGDR14	49.64	49.64	49.64	49.64	49.64	49.64	49.17	49.74
PkcPeriod02spat	25.00	25.00	25.00	25.00	25.00	25.00	25.00	25.00
PkcPeriod03spat	25.00	25.00	25.00	25.00	50.00	50.00	50.00	50.00
PkcPeriod04spat	40.00	40.00	40.00	40.00	40.00	40.00	40.00	40.00
PkcPeriod05spat	25.00	25.00	25.00	25.00	25.00	25.00	25.00	25.00
PkcPeriod06spat	40.00	40.00	40.00	20.00	40.00	40.00	20.00	20.00
PkcPeriod07spat	40.00	40.00	40.00	40.00	20.00	20.00	20.00	20.00
PkcPeriod08spat	20.00	20.00	20.00	40.00	40.00	40.00	40.00	40.00
PkcPeriod09spat	20.00	20.00	20.00	20.00	40.00	40.00	40.00	40.00
PkcPeriod10spat	20.00	20.00	20.00	20.00	40.00	40.00	40.00	40.00
PkcPeriod11spat	20.00	20.00	20.00	40.00	20.00	20.00	20.00	20.00
PkcPeriod12spat	20.00	40.00	40.00	40.00	20.00	20.00	20.00	20.00
PkcPeriod13spat	40.00	20.00	20.00	20.00	40.00	40.00	40.00	40.00
PkcPeriod14spat	20.00	20.00	20.00	40.00	40.00	40.00	40.00	40.00
PkcPeriod15spat	20.00	40.00	40.00	40.00	20.00	20.00	20.00	20.00
PkcPeriod16spat	40.00	40.00	40.00	20.00	40.00	40.00	20.00	20.00
PkcPiEN02spat	0.00	0.00	0.00	0.00	0.00	0.00	0.00	0.00
PkcPiEN03spat	0.00	0.00	0.00	0.00	2.56	2.56	2.39	2.39
PkcPiEN04spat	0.00	0.00	0.00	0.00	0.00	0.00	0.00	0.00
PkcPiEN05spat	0.00	0.00	0.00	0.00	0.00	0.00	0.00	0.00
PkcPiEN06spat	0.00	0.00	0.00	0.00	1.28	1.28	0.00	0.00

PkcPiEN07spat	0.00	0.00	0.00	0.00	0.00	0.00	0.00	0.00
PkcPiEN08spat	0.00	0.00	0.00	0.00	2.52	2.52	2.36	2.36
PkcPiEN09spat	0.00	0.00	0.00	0.00	2.52	2.52	2.35	2.35
PkcPiEN10spat	0.00	0.00	0.00	0.00	0.00	0.00	0.00	0.00
PkcPiEN11spat	0.00	0.00	0.00	0.00	0.00	0.00	0.00	0.00
PkcPiEN12spat	0.00	0.00	0.00	0.00	0.00	0.00	0.00	0.00
PkcPiEN13spat	0.00	0.00	0.00	0.00	3.63	3.63	0.00	0.00
PkcPiEN14spat	0.00	0.00	0.00	3.92	3.53	3.53	0.00	0.00
PkcPiEN15spat	0.00	3.88	1.27	0.00	0.00	0.00	0.00	0.00
PkcPiEN16spat	16.57	0.00	55.24	0.00	60.01	60.01	0.00	0.00
PkcSigmaEN02spat	66.92	66.92	66.93	66.93	67.11	67.11	67.20	67.20
PkcSigmaEN03spat	67.72	67.72	67.72	67.72	69.91	69.91	71.36	71.36
PkcSigmaEN04spat	80.10	80.10	80.52	80.52	82.58	82.58	80.52	80.52
PkcSigmaEN05spat	67.37	67.37	67.41	67.41	67.88	67.88	67.41	67.41
PkcSigmaEN06spat	79.95	79.05	81.87	79.37	85.80	85.80	78.92	78.92
PkcSigmaEN07spat	66.28	67.65	66.34	70.57	66.77	66.77	66.00	66.00
PkcSigmaEN08spat	66.29	67.02	66.15	66.60	67.82	67.82	69.41	69.41
PkcSigmaEN09spat	68.53	68.67	69.39	68.53	70.15	70.15	71.56	71.56
PkcSigmaEN10spat	67.04	67.04	67.72	67.04	72.61	72.61	69.35	69.35
PkcSigmaEN11spat	10.34	10.32	10.32	11.18	10.35	10.35	10.32	10.32
PkcSigmaEN12spat	73.73	79.00	78.01	81.92	73.89	73.89	73.75	73.75
PkcSigmaEN13spat	78.73	73.60	74.17	73.61	92.30	92.30	73.98	73.98
PkcSigmaEN14spat	70.62	69.98	69.98	84.55	81.53	81.53	70.78	70.78
PkcSigmaEN15spat	80.59	97.03	85.57	86.90	79.25	79.25	78.74	78.74
PkcSigmaEN16spat	81.28	81.28	93.34	74.51	90.03	90.03	74.52	74.52
PkcValenceelectrons02spat	14.29	14.29	14.29	14.29	14.29	14.29	14.29	14.29
PkcValenceelectrons03spat	14.29	14.29	14.29	14.29	57.14	57.14	57.14	57.14
PkcValenceelectrons04spat	57.14	57.14	57.14	57.14	57.14	57.14	57.14	57.14
PkcValenceelectrons05spat	14.29	14.29	14.29	14.29	14.29	14.29	14.29	14.29
PkcValenceelectrons06spat	57.14	57.14	57.14	14.29	71.43	71.43	14.29	14.29
PkcValenceelectrons07spat	57.14	57.14	57.14	57.14	14.29	14.29	14.29	14.29
PkcValenceelectrons08spat	14.29	14.29	14.29	57.14	57.14	57.14	57.14	57.14
PkcValenceelectrons09spat	14.29	14.29	14.29	14.29	57.14	57.14	57.14	57.14
PkcValenceelectrons10spat	14.29	14.29	14.29	14.29	57.14	57.14	57.14	57.14
PkcValenceelectrons11spat	14.29	14.29	14.29	57.14	14.29	14.29	14.29	14.29
PkcValenceelectrons12spat	14.29	57.14	57.14	71.43	14.29	14.29	14.29	14.29
PkcValenceelectrons13spat	57.14	14.29	14.29	14.29	85.71	85.71	57.14	57.14
PkcValenceelectrons14spat	14.29	14.29	14.29	57.14	57.14	57.14	57.14	57.14
PkcValenceelectrons15spat	14.29	85.71	71.43	85.71	14.29	14.29	14.29	14.29
PkcValenceelectrons16spat	71.43	71.43	85.71	14.29	57.14	57.14	14.29	14.29
PkcVdwradius02spat	64.86	64.86	64.86	64.86	64.86	64.86	64.86	64.86
PkcVdwradius03spat	57.14	57.14	57.14	57.14	80.95	80.95	80.95	80.95
PkcVdwradius04spat	80.95	80.95	80.95	80.95	80.95	80.95	80.95	80.95
PkcVdwradius05spat	57.14	57.14	57.14	57.14	57.14	57.14	57.14	57.14
PkcVdwradius06spat	80.95	80.95	80.95	57.14	73.81	73.81	57.14	57.14
PkcVdwradius07spat	80.95	80.95	80.95	80.95	57.14	57.14	57.14	57.14
PkcVdwradius08spat	57.14	57.14	57.14	80.95	80.95	80.95	80.95	80.95
PkcVdwradius09spat	57.14	57.14	57.14	57.14	80.95	80.95	80.95	80.95
PkcVdwradius10spat	57.14	57.14	57.14	57.14	80.95	80.95	80.95	80.95
PkcVdwradius11spat	57.14	57.14	57.14	80.95	57.14	57.14	57.14	57.14
PkcVdwradius12spat	57.14	80.95	80.95	73.81	57.14	57.14	57.14	57.14
PkcVdwradius13spat	80.95	57.14	57.14	57.14	72.38	72.38	80.95	80.95
PkcVdwradius14spat	57.14	57.14	57.14	80.95	80.95	80.95	80.95	80.95
PkcVdwradius15spat	57.14	72.38	73.81	72.38	57.14	57.14	57.14	57.14
PkcVdwradius16spat	73.81	73.81	72.38	57.14	80.95	80.95	57.14	57.14

PkHyb02spat	0.00	0.00	0.00	0.00	0.00	0.00	0.00	0.00
PkHyb03spat	0.00	0.00	0.00	0.00	100.00	100.00	100.00	100.00
PkHyb04spat	0.00	0.00	0.00	0.00	0.00	0.00	0.00	0.00
PkHyb05spat	0.00	0.00	0.00	0.00	0.00	0.00	0.00	0.00
PkHyb06spat	0.00	0.00	0.00	0.00	0.00	0.00	0.00	0.00
PkHyb07spat	0.00	0.00	0.00	0.00	0.00	0.00	0.00	0.00
PkHyb08spat	0.00	0.00	0.00	0.00	100.00	100.00	100.00	100.00
PkHyb09spat	0.00	0.00	0.00	0.00	100.00	100.00	100.00	100.00
PkHyb10spat	0.00	0.00	0.00	0.00	0.00	0.00	0.00	0.00
PkHyb11spat	0.00	0.00	0.00	0.00	0.00	0.00	0.00	0.00
PkHyb12spat	0.00	0.00	0.00	0.00	0.00	0.00	0.00	0.00
PkHyb13spat	0.00	0.00	0.00	0.00	100.00	100.00	0.00	0.00
PkHyb14spat	0.00	0.00	0.00	100.00	100.00	100.00	0.00	0.00
PkHyb15spat	0.00	100.00	0.00	0.00	0.00	0.00	0.00	0.00
PkHyb16spat	0.00	0.00	100.00	0.00	100.00	100.00	0.00	0.00
PPkcAromaticProt	0.00	0.00	0.00	0.00	0.00	0.00	0.00	0.00
PPkcAvhybridization	4.45	4.12	4.06	4.14	3.90	3.90	3.78	3.78
PPkcAvsigmaEN	45.20	49.16	48.67	47.92	52.72	52.72	48.47	48.47
PPkcAvvalenceelectrons	15.69	19.44	19.44	19.75	33.33	33.33	25.00	25.00
PPkcConjugatedpiProt	0.00	0.00	0.00	0.00	0.00	0.00	0.00	0.00
PPkcEffpolarProt	13.52	13.52	13.87	13.85	19.48	19.48	20.55	20.55
PPkcHybConn	0.00	0.00	0.00	0.00	0.00	0.00	0.00	0.00
PPkcPichargeConn	4.21	4.21	4.21	4.21	4.21	4.21	4.21	4.21
PPkcPichargemax2sph	0.00	0.00	0.00	0.00	0.00	0.00	0.00	0.00
PPkcPichargemin2sph	79.76	79.76	79.76	79.76	79.76	79.76	79.76	79.76
PPkcPieENConn	0.00	0.00	0.00	0.00	0.00	0.00	0.00	0.00
PPkcPiEN2sph	0.00	0.00	0.00	0.00	2.56	2.56	2.39	2.39
PPkcPiEN2sphmax	0.00	0.00	0.00	0.00	2.56	2.56	2.39	2.39
PPkcPiEN2sphmin	0.00	0.00	0.00	0.00	0.00	0.00	0.00	0.00
PPkcSigmachargeaverage2sph	19.70	19.70	19.74	19.74	19.87	19.87	19.53	19.53
PPkcSigmachargemax2sph	0.08	0.08	0.08	0.08	0.17	0.17	0.10	0.10
PPkcSigmachargemin2sph	25.07	25.07	25.13	25.13	25.31	25.31	24.95	24.95
PPkcSigmachargeProt	50.16	50.16	50.18	50.18	52.58	52.58	53.65	53.65
PPkcSigmaEN01spat	91.19	91.19	91.19	91.19	91.62	91.62	91.81	91.81
PPkcSigmaEN2sphmax	50.07	50.07	51.09	51.09	55.98	55.98	54.21	54.21
PPkcSigmaEN2sphmin	86.71	86.71	86.71	86.71	86.81	86.81	86.86	86.86
PPkcSigmaENConn	49.02	49.02	49.11	49.11	51.12	51.12	51.13	51.13
PPkcTotalchargeaverage2sph	71.32	71.32	71.32	71.32	71.33	71.33	71.30	71.30
PPkcTotalchargemin2sph	0.04	0.04	0.04	0.04	0.10	0.10	0.06	0.06
PPkcTotalchargeProt	50.16	50.16	50.18	50.18	52.58	52.58	53.65	53.65
PPkcTotchargeConn	9.08	9.08	9.08	9.08	9.14	9.14	9.14	9.14
PPpkcPichargeaverage2sph	72.43	72.43	72.43	72.43	72.43	72.43	72.43	72.43
PPpkcSigmachargeConn	93.70	93.70	93.73	93.73	94.30	94.30	94.31	94.31
PPPkcTotalchargemax2sph	79.84	79.84	79.84	79.84	79.86	79.86	79.83	79.83
PSpatAvrepdistance	58.20	62.09	61.46	59.71	61.89	61.89	62.88	62.88
PTopoCountcTwobaway	33.33	33.33	33.33	33.33	66.67	66.67	66.67	66.67
PTopoCountoTwoaway	0.00	0.00	0.00	0.00	0.00	0.00	0.00	0.00
PTopoSubstituentsConnected	33.33	33.33	33.33	33.33	66.67	66.67	66.67	66.67
SpaMindistToHy02spat	0.00	0.00	0.00	0.00	0.00	0.00	0.00	0.00
SpaMindistToHy03spat	0.00	0.00	0.00	0.00	57.74	57.74	57.74	57.74
SpaMindistToHy04spat	46.38	46.38	46.38	46.38	46.38	46.38	46.05	46.05
SpaMindistToHy05spat	52.63	52.62	52.62	51.94	53.62	53.62	47.13	47.13
SpaMindistToHy06spat	51.79	51.79	51.79	33.57	50.39	50.39	42.49	42.49
SpaMindistToHy07spat	45.96	45.96	45.96	45.26	41.25	41.25	33.44	33.44
SpaMindistToHy08spat	38.80	29.55	38.78	42.41	40.73	40.73	41.54	41.54

SpaMindistToHy09spat	36.85	36.22	30.34	37.57	40.06	40.06	48.01	48.01
SpaMindistToHy10spat	30.73	30.73	30.68	30.19	33.70	33.70	33.60	33.60
SpaMindistToHy11spat	29.68	29.13	29.70	28.61	30.21	30.21	30.26	30.26
SpaMindistToHy12spat	42.07	29.40	29.78	29.37	26.29	26.29	22.81	22.81
SpaMindistToHy13spat	43.15	38.85	30.70	19.67	36.61	36.61	26.50	26.50
SpaMindistToHy14spat	42.40	36.07	35.84	29.06	31.77	31.77	24.02	24.02
SpaMindistToHy15spat	37.96	25.46	38.31	27.36	30.61	30.61	24.31	24.31
SpaMindistToHy16spat	39.37	29.20	38.84	33.32	29.61	29.61	26.60	26.60
SpatAvdistohy03spat	32.10	32.10	32.10	32.10	57.74	57.74	57.80	57.80
SpatAvdistohy04spat	46.38	46.38	46.38	46.38	46.38	46.38	46.11	46.11
SpatAvdistohy05spat	56.68	56.68	56.68	56.68	59.28	59.28	53.31	53.31
SpatAvdistohy06spat	56.35	56.35	56.35	52.00	57.50	57.50	48.34	48.34
SpatAvdistohy07spat	50.01	50.01	50.01	50.01	41.70	41.70	45.95	45.95
SpatAvdistohy08spat	48.20	43.84	48.18	46.86	46.22	46.22	46.60	46.60
SpatAvdistohy09spat	45.67	45.00	43.81	45.68	44.98	44.98	48.45	48.45
SpatAvdistohy10spat	38.08	38.08	37.99	38.07	33.90	33.90	38.08	38.08
SpatAvdistohy11spat	36.65	36.10	36.66	37.41	36.89	36.89	37.33	37.33
SpatAvdistohy12spat	44.16	37.44	37.80	36.70	33.24	33.24	31.84	31.84
SpatAvdistohy13spat	45.21	41.03	38.23	30.98	39.32	39.32	33.51	33.51
SpatAvdistohy14spat	44.58	37.87	37.85	34.72	35.86	35.86	30.46	30.46
SpatAvdistohy15spat	43.27	34.91	39.94	33.65	32.78	32.78	30.94	30.94
SpatAvdistohy16spat	42.74	36.12	42.44	35.27	33.66	33.66	30.33	30.33
SpatAvdisttohy01spat	99.99	99.99	99.99	99.99	75.00	75.00	72.56	72.56
SpatAvdisttohy02spat	46.61	46.61	46.61	46.61	34.96	34.96	33.82	33.82
SpatDistancetoatom02spat	55.44	55.44	55.44	55.44	55.44	55.44	56.13	56.13
SpatDisttoatom01spat	94.78	94.78	94.78	94.78	94.78	94.78	95.84	95.84
SpatDisttoatom03spat	41.20	41.19	41.20	41.20	56.95	56.95	56.13	56.13
SpatDisttoatom04spat	42.14	42.15	42.14	42.15	42.15	42.15	41.81	41.81
SpatDisttoatom05spat	52.82	52.83	52.83	52.83	53.15	53.15	52.54	52.54
SpatDisttoatom06spat	54.81	54.81	54.81	54.72	54.12	54.12	47.08	47.08
SpatDisttoatom07spat	51.06	51.06	51.06	51.06	51.04	51.04	49.73	49.73
SpatDisttoatom08spat	50.83	48.53	50.81	47.29	47.38	47.38	46.06	46.06
SpatDisttoatom09spat	43.18	42.54	42.67	43.18	39.65	39.65	40.25	40.25
SpatDisttoatom10spat	38.45	38.45	38.39	38.45	35.63	35.63	36.71	36.71
SpatDisttoatom11spat	36.89	36.33	36.90	38.64	36.00	36.00	36.11	36.11
SpatDisttoatom12spat	42.70	36.98	37.38	36.17	33.47	33.47	33.77	33.77
SpatDisttoatom13spat	44.81	40.11	39.79	34.28	35.60	35.60	32.94	32.94
SpatDisttoatom14spat	44.74	37.29	37.27	36.65	33.42	33.42	30.72	30.72
SpatDisttoatom15spat	43.94	37.62	39.63	35.51	34.12	34.12	33.63	33.63
SpatDisttoatom16spat	41.95	35.81	41.73	33.67	32.92	32.92	32.59	32.59
SpatGasteigerG3R00	52.06	52.06	52.06	52.06	52.06	52.06	48.98	49.10
SpatGasteigerG3R01	48.66	48.66	48.66	48.66	48.66	48.66	44.88	45.08
SpatGasteigerG3R02	36.50	36.50	36.50	36.50	36.50	36.50	47.30	47.31
SpatGasteigerG3R03	49.56	49.56	49.56	49.56	49.56	49.56	61.28	61.28
SpatGasteigerG3R04	84.09	84.09	84.09	84.09	84.09	84.09	90.03	90.03
SpatGasteigerG3R05	92.87	92.87	92.87	92.87	92.87	92.87	94.27	94.26
SpatGasteigerG3R06	92.85	92.85	92.85	92.85	92.85	92.85	92.68	92.67
SpatGasteigerG3R07	86.52	86.52	86.52	86.52	86.52	86.52	84.97	84.97
SpatGasteigerG3R08	84.89	84.89	84.89	84.89	84.89	84.89	82.82	82.85
SpatGasteigerG3R09	59.92	59.92	59.92	59.92	59.92	59.92	58.24	58.28
SpatGasteigerG3R10	51.04	51.04	51.04	51.04	51.04	51.04	49.93	49.94
SpatGasteigerG3R11	28.09	28.09	28.09	28.09	28.09	28.09	21.60	21.63
SpatGasteigerG3R12	64.14	64.14	64.14	64.14	64.14	64.14	60.72	60.76
SpatGasteigerG3R13	86.18	86.18	86.18	86.18	86.18	86.18	87.00	86.99
SpatGasteigerG3R14	87.57	87.57	87.57	87.57	87.57	87.57	87.76	87.75

SpatGasteigerGSR00	39.90	39.90	39.90	39.90	39.90	39.90	54.48	55.10
SpatGasteigerGSR01	38.55	38.55	38.55	38.55	38.55	38.55	55.82	55.29
SpatGasteigerGSR02	36.39	36.39	36.39	36.39	36.39	36.39	55.49	53.93
SpatGasteigerGSR03	34.10	34.10	34.10	34.10	34.10	34.10	54.36	51.90
SpatGasteigerGSR04	31.99	31.99	31.99	31.99	31.99	31.99	52.91	49.69
SpatGasteigerGSR05	30.08	30.08	30.08	30.08	30.08	30.08	51.25	47.44
SpatGasteigerGSR06	28.40	28.40	28.40	28.40	28.40	28.40	49.50	45.29
TopoBondsToAtom03spat	20.00	20.00	20.00	20.00	20.00	20.00	20.00	20.00
TopoBondsToAtom04spat	10.00	10.00	10.00	10.00	10.00	10.00	10.00	10.00
TopoBondsToAtom05spat	18.18	18.18	18.18	18.18	18.18	18.18	18.18	18.18
TopoBondsToAtom06spat	11.77	11.77	11.77	23.53	11.77	11.77	11.77	11.77
TopoBondsToAtom07spat	7.41	7.41	7.41	7.41	11.11	11.11	14.81	14.81
TopoBondsToAtom08spat	15.79	21.05	15.79	10.53	10.53	10.53	10.53	10.53
TopoBondsToAtom09spat	15.79	15.79	21.05	15.79	10.53	10.53	10.53	10.53
TopoBondsToAtom10spat	16.67	16.67	16.67	16.67	11.11	11.11	11.11	11.11
TopoBondsToAtom11spat	11.54	11.54	11.54	11.54	11.54	11.54	15.38	15.38
TopoBondsToAtom12spat	11.54	11.54	11.54	11.54	11.54	11.54	15.38	15.38
TopoBondsToAtom13spat	11.11	11.11	14.81	29.63	14.81	14.81	11.11	11.11
TopoBondsToAtom14spat	15.38	11.54	11.54	15.38	11.54	11.54	11.54	11.54
TopoBondsToAtom15spat	19.23	23.08	11.54	15.38	11.54	11.54	15.38	15.38
TopoBondsToAtom16spat	15.38	15.38	19.23	11.54	11.54	11.54	15.38	15.38
TopoGasteigerGHRtopol00	0.00	0.00	0.00	0.00	0.00	0.00	19.04	17.93
TopoGasteigerGHRtopol01	0.00	0.00	0.00	0.00	0.00	0.00	21.30	22.34
TopoGasteigerGHRtopol02	0.00	0.00	0.00	0.00	0.00	0.00	21.53	24.92
TopoGasteigerGHRtopol03	0.00	0.00	0.00	0.00	0.00	0.00	19.80	25.06
TopoGasteigerGHRtopol04	0.00	0.00	0.00	0.00	0.00	0.00	17.90	24.56
TopoGasteigerGHRtopol05	0.00	0.00	0.00	0.00	0.00	0.00	12.55	18.54
TopoGasteigerGHRtopol06	0.00	0.00	0.00	0.00	0.00	0.00	8.42	13.31
TopoPicontact01spat	0.00	0.00	0.00	0.00	0.00	0.00	0.00	0.00
TopoPicontact02spat	0.00	0.00	0.00	0.00	0.00	0.00	0.00	0.00
TopoPicontact03spat	0.00	0.00	0.00	0.00	100.00	100.00	0.00	0.00
TopoPicontact04spat	0.00	0.00	0.00	0.00	0.00	0.00	0.00	0.00
TopoPicontact05spat	0.00	0.00	0.00	0.00	0.00	0.00	0.00	0.00
TopoPicontact06spat	0.00	0.00	0.00	0.00	0.00	0.00	0.00	0.00
TopoPicontact07spat	0.00	0.00	0.00	0.00	0.00	0.00	0.00	0.00
TopoPicontact08spat	0.00	0.00	0.00	0.00	100.00	100.00	0.00	0.00
TopoPicontact09spat	0.00	0.00	0.00	0.00	100.00	100.00	0.00	0.00
TopoPicontact10spat	0.00	0.00	0.00	0.00	0.00	0.00	0.00	0.00
TopoPicontact11spat	0.00	0.00	0.00	0.00	100.00	100.00	0.00	0.00
TopoPicontact12spat	0.00	0.00	0.00	0.00	100.00	100.00	0.00	0.00
TopoPicontact13spat	0.00	0.00	0.00	0.00	0.00	0.00	0.00	0.00
TopoPicontact14spat	0.00	0.00	0.00	0.00	0.00	0.00	0.00	0.00
TopoPicontact15spat	0.00	0.00	0.00	0.00	0.00	0.00	0.00	0.00
TopoPicontact16spat	0.00	0.00	0.00	0.00	0.00	0.00	0.00	0.00
TopoProtoncategory	100.00	100.00	100.00	100.00	100.00	100.00	75.00	75.00

Table 2: complete Descriptor set for given atomIDs (hard errors)

Loadings

The component loadings of the top 5 of our descriptors are shown in fig. 1.

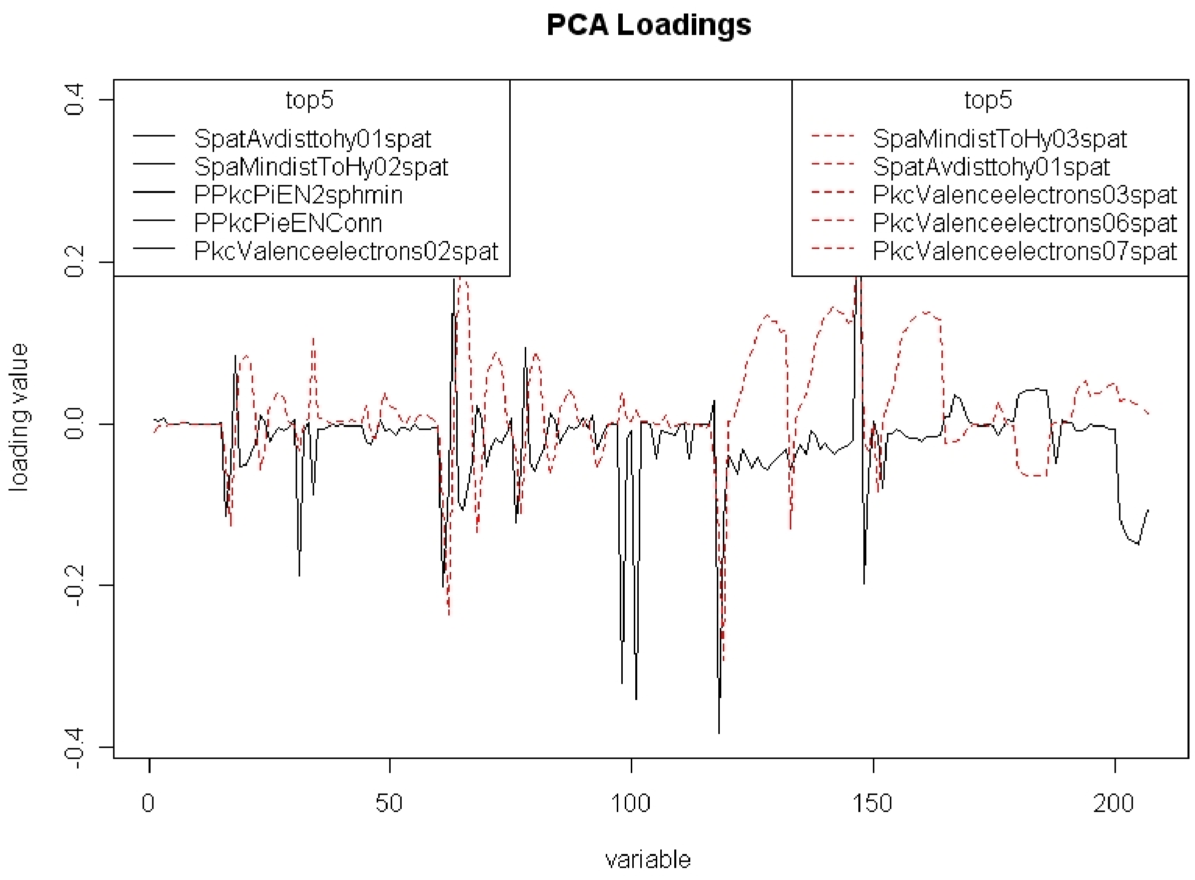

Figure 1: The component loadings of the top 5 of our descriptors.

References

[1] Aires-De-Sousa J, Hemmer M, Gasteiger J: **Prediction of 1H-NMR Chemical Shifts Using Neural Networks**. *Anal. Chem.* 2002, **74**:80–90.

[2] Meiler J: **PROSHIFT: Protein chemical shift prediction using artificial neural networks**. *J. of Biomolecular NMR* 2003, **26**:25–37.
